# Supplementary material for: The genome sequence of Cardamine flexuosa With., 1796 (Brassicales: Brassicaceae)
Source: Wellcome Open Res. 2026 May 26;11:300. [Version 1] doi: 10.12688/wellcomeopenres.26645.1 (PMC13555072; doi:10.12688/wellcomeopenres.26645.1)
Supplement: Peer review history

# Reviewer report: Reviewer response for version 1

**DOI:** 10.21956/wellcomeopenres.29342.r158146

**Published:** 2026-07-13

**Authors:** Osamah Alisawi

**Affiliations:** 1 University of Kufa, Kufa, Iraq

**Review comments:**

**Dear Editor**

**I sincerely appreciate your confidence in my ability to evaluate this MS. Please find my general remarks below:**

**Assessment and Summary:**

 The manuscript presents a high-quality, chromosome-level reference genome assembly for Cardamine flexuosa (Wavy Bitter-cress). The work was conducted under the umbrella of the Darwin Tree of Life project, which intends to catalog eukaryotic biodiversity across Britain and Ireland. As a Data Note submitted to Wellcome Open Research, the scope of the manuscript is deliberately limited to a detailed description of the dataset, its protocol, validation metrics, and deposition pathways without exhaustive biological analysis or interpretation. The execution of the multi-omics strategy combining PacBio HiFi sequencing, Illumina Hi-C scaffolding, and RNA-seq is sound and highly reproducible. The primary assembly spans 204.54 Mb across 8 chromosomal pseudomolecules, capturing 97.53% of the genomic sequence. With a high Consensus Quality score (QV 61.8) and strong proteome completeness (98.4% BUSCO), this dataset represents a pristine, utility-driven resource for plant evolutionary biology. The manuscript is exceptionally well-structured and maps clearly to the platform's criteria. I recommend approving this manuscript (Verdict: Approved), subject to addressing a few minor structural clarifications.

 General Feedback

- Methods & Protocols

For DNA and RNA extractions, sample weight needs to be specified.

  Strong documentation of the collection metadata includes precise geographic coordinates, tissue provenance (leaf), and voucher identification (ddCarFlex1) (p. 3). An appropriate validation step is to use flow cytometry as an orthogonal method to estimate the 1C-value genome size (~250 Mb) before parsing sequencing methods (pp. 3, 6).

  The authors state on page 3 that they use a Covaris g-TUBE to fragment the collected high molecular weight (HMW) DNA to the 8–10 kb range. However, they claim that "the DNA was fragmented to ~10 kb" (p. 4) before library preparation. If this was a single fragmentation procedure or a secondary refinement step, authors should make that clear.

 Why the authors used both platforms instead of just one of Illumina ( NovaSeq 6000 and NovaSeq X) for sequencing in order to prepare DNA and RNA libraries, respectively.

 I am aware that the study focused on a data note, but it is crucial to address repeating regions or at the very least highlight the ratio and key elements of the repeats. This task might be completed with a single RepeatExplorer pipeline run.

 - Bioinformatics & Assembly Quality

  

- The software pipeline complies with the most recent *de novo* plant genomics gold standards. It is best to use OATK for isolated organelle parsing, YaHS for structural Hi-C scaffolding, and Hifiasm for primary block curation (p. 5). Appropriate post-assembly stringency is demonstrated by manual curation using PretextView, which involves two breaks and eleven joins (p. 5).

- The authors state that the alternate haplotype assembly was also deposited in INSDC databases but note its k-mer completeness is quite low at 1.55% (compared to 99.55% for the primary assembly) (pp. 8, 10). Given that GenomeScope2.0 computed a very low heterozygosity level of 0.01%, this drop-off is mathematically expected (p. 6). Adding one sentence linking this low alternate haplotype recovery directly to the homozygosity of the plant would aid readers who look at Table 4 (p. 10).

Are sufficient details of methods and materials provided to allow replication by others?

Yes

Is the rationale for creating the dataset(s) clearly described?

Yes

Are the datasets clearly presented in a useable and accessible format?

Yes

Are the protocols appropriate and is the work technically sound?

Yes

Reviewer Expertise:

My areas of interest include genome analysis and bioinfomatics tools to take into account high quality data assembly, particularly repetitive regions, integrated viruses, and specific gene annotations, as well as high throughput sequencing, particularly short read output in Illumina platforms.

I confirm that I have read this submission and believe that I have an appropriate level of expertise to confirm that it is of an acceptable scientific standard.

# Reviewer report: Reviewer response for version 1

**DOI:** 10.21956/wellcomeopenres.29342.r156820

**Published:** 2026-07-10

**Authors:** Rohit Joshi

**Affiliations:** 1 CSIR-Institute of Himalayan Bioresource Technology, Palampur, India

The authors present a chromosome-level genome assembly of *Cardamine flexuosa* generated using PacBio HiFi long-read sequencing, Hi-C scaffolding, and RNA-seq-assisted annotation. The assembly demonstrates excellent technical quality, with 97.53% of the genome anchored to eight chromosomes, high sequence completeness (BUSCO 98.4%), high consensus quality (QV 59.1), and comprehensive gene annotation comprising 24,305 protein-coding genes. The study represents a valuable genomic resource for the Brassicaceae community and contributes to the objectives of the Darwin Tree of Life Project. The Discussion section is brief and emphasizes mainly on assembly statistics.

 1- The main part, i.e., biological interpretation of this assembly or what biological study as outcome besides sequence resource.

 2- Can any evolutionary gap be retrieved?

Are sufficient details of methods and materials provided to allow replication by others?

Yes

Is the rationale for creating the dataset(s) clearly described?

Yes

Are the datasets clearly presented in a useable and accessible format?

Yes

Are the protocols appropriate and is the work technically sound?

Yes

Reviewer Expertise:

Functional Genomics

I confirm that I have read this submission and believe that I have an appropriate level of expertise to confirm that it is of an acceptable scientific standard.
